# The relationship between age and suicidal thoughts and attempted suicide among prisoners

**DOI:** 10.1186/s40352-020-00117-3

**Published:** 2020-06-22

**Authors:** Bryce E. Stoliker, Simon N. Verdun-Jones, Adam D. Vaughan

**Affiliations:** 1 School of Criminology, Simon Fraser University, Burnaby, British Columbia V5A 1S6 Canada; 2 School of Criminal Justice, Texas State University, San Marcos, TX 78666 USA

**Keywords:** Suicidal thoughts, Attempted suicide, Older prisoners, Younger prisoners, Lifespan developmental theory, Aging

## Abstract

**Background:**

Suicide is a major problem across the lifespan, yet rates are highest among middle-aged and older adults; a trend which remains relatively stable across varying sociological settings, including prisons. Despite this understanding, there is limited knowledge on the nature of suicidal thoughts and attempts among older prisoners, especially with respect to how they compare to younger counterparts. The present study aimed to increase insight into the relationship between age and suicidal thoughts and attempted suicide among prisoners, with particular focus on factors that may explain age-based variability.

**Results:**

Cross-sectional data were drawn from a nationally representative sample of 18,185 prisoners housed within 326 prisons across the United States. In general, analyses revealed that: (a) attempted suicide was more commonly reported among younger prisoners, while suicidal ideation was more commonly reported among older prisoners; (b) the relationship between age and probability of reporting suicidal thoughts and behavior is curvilinear; (c) younger and older prisoners exhibit somewhat differing predictive patterns of suicidal thoughts and behavior (e.g., physical illness is directly associated with suicidal history for younger prisoners, whereas the effect of physical illness on suicidal history for older prisoners is mediated by depression).

**Conclusions:**

There is evidence to suggest that suicidal thoughts and behavior may manifest differently for younger and older prisoners, with differing patterns of risk. More research is needed on age-based variability in suicidal thoughts and attempted suicide among prisoners, as well as those factors that might explain this variability. Importantly, future research must continue to investigate the nature of suicidal thoughts and behavior among older prisoners.

## Background

Suicide is a major problem across the lifespan; yet, internationally, rates are consistently highest among middle-aged and older adults (see Fiske & O’Riley, 2016; Lutz, Morton, Turiano, & Fiske, 2016; Stanley, Hom, Rogers, Hagan, & Joiner, 2016; World Health Organization (WHO), 2014), which is a trend that remains relatively stable across differing sociological settings (see Stanley et al., 2016; see also Nock et al., 2008; World Health Organization (WHO), 2014). Indeed, in the United States, older prisoners exhibit the highest rates of suicide in correctional systems (Barry, Wakefield, Trestman, & Conwell, 2017; Carson & Cowhig, 2020; Noonan, Rohloff, & Ginder, 2015). Despite that suicide in later life has become a major public health issue, there has been limited scholarly and public attention devoted to suicidality among older adults (Lutz et al., 2016; Van Orden & Deming, 2018). Much less is known about the nature of suicidal thoughts and attempts among older prisoners (see Barry et al., 2017), especially with respect to how older prisoners compare to younger counterparts.

It has been suggested that late-life suicidal ideation and behavior differs in important ways from suicidal ideation and behavior earlier in the lifespan (Fiske & O’Riley, 2016; O’Riley, Van Orden, & Conwell, 2014; see also Lutz et al., 2016; Stanley et al., 2016; Van Orden & Conwell, 2016; Van Orden & Deming, 2018), which necessitates the consideration of age-focused analyses on suicide. With the global trend towards an aging population, it is expected that numbers of suicide will increase (Lutz et al., 2016) and suicide in later life will be an increasing problem (Van Orden & Deming, 2018). In the same way, population aging is taking place within prison systems in high-income countries (see Barry et al., 2017; Blowers & Blevins, 2015; Kakoullis, Le Mesurier, & Kingston, 2010; Opitz-Welke et al., 2019; Stoliker & Galli, 2019) and, therefore, it is expected that these facilities will face increasing challenges surrounding suicidal thoughts and behavior among the older inmate population (Barry et al., 2017). To that end, the purpose of the current study was to investigate suicidal thoughts and attempted suicide among prisoners from a theoretical and methodological perspective that intends to increase insight into the relationship between age and suicidality.

### Defining the ‘older’ prisoner

There is no question that what qualifies an individual as ‘older’ or ‘aging’ is specific to the context and population (Uzoaba, 1998). In the context of prisons, there had been inconsistencies with respect to what defines an ‘older’ inmate and, therefore, disagreement in the age-threshold that ought to be used to distinguish younger from older prisoners (Aday, 1994; Grant, 1999; Uzoaba, 1998). However, most commonly, older prisoners have been classified as aged 50 years and older (Grant, 1999; Horowitz, 2013; Morton, 1992; Opitz-Welke et al., 2019; Stoliker & Galli, 2019; Stoliker & Varanese, 2017; Uzoaba, 1998). Though a 50-year-old might still be considered fairly young by contemporary standards, the use of such a low age-threshold for studying prison populations is primarily justified in the fact that prisoners often display an ‘accelerated’ physical age, experiencing physiological disease/illness much earlier in the lifespan than non-incarcerated persons—owing to the lifestyles/adversities of pre-prison and prison life (Barry et al., 2017; Grant, 1999; Kratcoski & Pownall, 1989; Maschi, Viola, & Sun, 2013; Reviere & Young, 2004; Williams et al., 2006). Accordingly, for the purposes of this study, ‘older prisoners’ refers to those aged 50 years and older, whereas ‘younger prisoners’ applies broadly to include those aged 49 years and younger.

### Theoretical perspective

The lifespan developmental theory (Fiske & O’Riley, 2016) provides a suitable framework for understanding why suicidal thoughts and behavior may vary across age. This theory posits that late-life suicidal ideation is linked to restrictions and adversities associated with aging—such as physical illnesses, cognitive impairment, interpersonal losses, and other age-related changes—whereby those who are unable to cope with, and adapt to, life’s changes will be at greater risk for suicidal ideation. In other words, a desire to die will manifest when an individual is unable to effectively adapt to changes associated with the aging process (e.g., not adopting strategies that compensate for age-related adversities and limitations). Indeed, it has been shown that older individuals with personality traits akin to a low “openness to experience” are at increased risk of suicide, perhaps reflecting individuals who are unable to cope with, and adapt to, challenges associated with aging (see Conwell, Duberstein, & Caine, 2002; Duberstein, Conwell, & Caine, 1994). Aligned with the interpersonal-psychological (Joiner, 2005; Van Orden et al., 2010) and 3-step (Klonsky & May, 2015) theories of suicide, those with a desire to die owing to the inability to cope with, or adapt to, life’s changes have the potential to enact suicidal behavior if they have an acquired capability for suicide (i.e., a lowered fear of death and higher pain tolerance) (Fiske & O’Riley, 2016). With Fiske and O’Riley’s (2016) lifespan developmental perspective in mind, it stands to reason that age-related adversities and limitations may distinguish older and younger prisoners who experience suicidal thoughts and/or attempt suicide.

### Age and Suicidality

Research has highlighted that individuals aged 60 and older are among the highest risk of suicide-related death in the general (i.e., non-incarcerated) population in the United States (see Fiske & O’Riley, 2016; Lutz et al., 2016; Stanley et al., 2016; Van Orden & Conwell, 2016), while individuals aged 50 and older are among the highest risk of suicide-related death in U.S. correctional facilities (Barry et al., 2017). It is apparent that the lethality of suicidal thoughts and behaviors in later life (Stanley et al., 2016) contributes to the high rate of suicide among older adults, as estimates from non-incarcerated populations suggest that the ratio of attempted to completed suicide is 4:1 among older adults (Conwell, 2013; Conwell et al., 2002) and 25:1 among younger adults (Institute of Medicine, 2002). The disparity in lethality of suicide across age has been linked to the fact that older adults often choose more violent methods (Conwell et al., 2002), have greater determination to die from an attempt (Conwell et al., 1998), and present fewer opportunities to be rescued owing to physical frailty (Stanley et al., 2016). In addition to these facts, it has been suggested that older adults are also less likely to express/report suicidal ideation prior to their deaths (Fiske & O’Riley, 2016), increasing the challenge of intervening and preventing advancement to suicidal behavior. Indeed, Lutz et al. (2016) suggest that suicidal ideation is less commonly reported among older persons.

Several factors have been identified as important correlates of suicidal thoughts and behavior among non-incarcerated older adults, which may help to distinguish risk of suicide later as compared to earlier in the lifespan. For instance, suicide in later life has been linked to personality traits that reflect a lack of flexibility (see Conwell et al., 2002; Fiske & O’Riley, 2016), social disconnectedness (Duberstein et al., 2004, b; Fässberg et al., 2012), physical health problems (Duberstein, Conwell, Conner, Eberly, & Caine, 2004; Erlangsen, Vach, & Jeune, 2005; Fässberg et al., 2016; Lutz et al., 2016), disabilities in basic activities of daily living (Conwell et al., 2010; Dennis et al., 2009; Fässberg et al., 2016), and sleep patterns (Bernert, Turvey, Conwell, & Joiner, 2014; Nadorff, Fiske, Sperry, Petts, & Gregg, 2013; Ross, Bernstein, Trent, Henderson, & Paganini-Hill, 1990). With respect to psychopathology, it has been suggested that affective psychiatric illnesses, such as depression, are common in suicide among older adults (Conwell et al., 2002; Conwell, Olsen, Caine, & Flannery, 1991; Van Orden & Deming, 2018), and that depression is more common among older suicide victims as compared to younger counterparts (see Conwell et al., 2002). Relatedly, a series of studies have shown that depression is the most commonly reported mental health problem among older prisoners (see Stoliker & Galli, 2019), which might suggest a point of therapeutic intervention for this population. Above all, Van Orden and Deming (2018) highlight that ageism—characterized by negative perceptions of aging and discriminatory behaviors toward older adults—may be the driving force behind the high rates of suicide among older and aging adults; accordingly, ageism could be directly or indirectly linked to the constellation of factors (such as those highlighted above) which characterize older individuals who die by suicide.

Given the prominence of physical illnesses and disabilities later in life, health conditions have received considerable attention as predictors of suicidal thoughts and behavior among older adults (Conwell et al., 2002). Lutz et al. (2016) showed that, among a sample of adults aged 50 years and older, several health conditions were linked to increased risk of suicidal ideation and that depression and disability mediated the association between health conditions and suicidal ideation (Lutz et al., 2016). Nevertheless, just because physical illnesses and disabilities are generally more prominent in later life does not necessarily mean that health conditions differentiate risk of suicide later as compared to earlier in the lifespan. Indeed, Scott et al. (2010) present evidence to suggest that illnesses were more strongly associated with suicidal ideation for younger as compared to older adults. In line with the lifespan developmental theory (Fiske & O’Riley, 2016), perhaps in some cases the inability to cope with, and adapt to, certain life changes is more pronounced earlier in the lifespan. For instance, a restriction or adversity associated with aging, such as physical disease/illness, developed earlier in the lifespan might have a more profound effect on an individual as compared to the development of such a restriction/adversity later in the lifespan. This is particularly relevant in the context of prison populations, as prisoners may experience physical disease/illness earlier in the lifespan than what is typically expected of the general public (Barry et al., 2017; Grant, 1999; Kratcoski & Pownall, 1989; Maschi et al., 2013; Reviere & Young, 2004; Williams et al., 2006).

Despite a growing body of literature focused on suicide among older and aging adults, along with the trend toward an aging prison population, there is a marked lack of empirical research on the nature of suicidal thoughts and behavior among older prisoners (e.g., see Barry et al., 2017; Opitz-Welke et al., 2019). Studies have, in some capacity, considered the relationship between age and suicidal thoughts/behavior among prisoners (e.g., see Blaauw, Winkel, & Kerkhof, 2001; Dye, 2010; Favril, Stoliker, & Vander Laenen, 2020; Marzano, Hawton, Rivlin, & Fazel, 2011; Stoliker, 2018), but this research has produced mixed findings. Some evidence suggests that prisoners who engage in suicidal behavior tend to be younger (Blaauw et al., 2001; Daniel & Fleming, 2006; Liebling, 1999; Marzano et al., 2011) and that an increase in age corresponds to decreased odds of reporting a previous suicide attempt compared to no attempt (Stoliker, 2018) and suicidal ideation only (Favril et al., 2020). Other researchers have reported a higher prevalence of suicidal behavior in older inmates (Blaauw, Kerkhof, & Hayes, 2005). Still, others have shown null patterns for age and suicide (e.g., see Dye, 2010; Green, Kendall, Andre, Looman, & Polvi, 1993; Mumola, 2005). Barry et al. (2017) and Opitz-Welke et al. (2019) provide the only known published research that specifically analyzes suicidal thoughts and behavior among older prisoners. In the former study, researchers examined the effects of disability in ‘prison activities of daily living’ (PADL) on suicidal ideation in a sample of 167 prisoners aged 50 years and older, finding that those who experienced disability in PADL were at increased likelihood of also experiencing suicidal ideation (Barry et al., 2017). In the latter study, the authors reported that, in German prisons, suicide rates are greatest among older prisoners and that this pattern remained stable for over a decade despite a decline in suicide rates for all ages (Opitz-Welke et al., 2019). Taken together, the relationship between age and suicidal thoughts and behavior among prison populations is still unclear, especially with respect to the nature of suicidal thoughts and behavior among older prisoners specifically.

### The current study

Given that researchers have signified there are important differences in suicidal thoughts and behavior later as compared to earlier in the lifespan (Fiske & O’Riley, 2016; O’Riley et al., 2014; see also Lutz et al., 2016; Stanley et al., 2016; Van Orden & Conwell, 2016; Van Orden & Deming, 2018), it is imperative that studies on prisoner suicidality extend beyond merely assigning age as a control or descriptive measure (e.g., see Barry et al., 2017). As such, the primary aim of this study was to elucidate the relationship between age and suicidal thoughts and attempts among prisoners, with particular focus on factors that may explain age-based variation in suicidality. It was hypothesized that: (a) incidence of suicidal thoughts and attempts will be greater among older compared to younger prisoners, and; (b) any age-based variation in suicidality will be a function of factors that are expected to distinguish suicidal thoughts and behavior later as compared to earlier in life, such as hopelessness (a component of depression, but regarded as a better predictor of suicidal thoughts and behavior: Beck, 1986; see also Stoliker, Verdun-Jones, & Vaughan, 2020), physical disease/illness, and disabilities in basic activities of daily living.

## Method

### Data

Data for this study came from the 2004 *Survey of Inmates in State and Federal Correctional Facilities* (SISFCF). This cross-sectional survey collected data from a nationally representative sample of 14,297 male and 3888 female prisoners in 326 U.S. prisons (287 state and 39 federal facilities). For reference, during the data collection period (2003–2004), U.S. state and federal prisons housed between 1.38 and 1.42 million prisoners (Harrison & Beck, 2004, 2005). A two-stage, multi-level sampling procedure was used to obtain the sample—whereby prison facilities were selected in the first stage and individual prisoners were selected in the second stage—and computer-assisted personal interviewing was used to gather self-report information from prisoners. For further detail on the nature of the survey and data collection, see the publicly available 2004 SISFCF codebook and dataset (U.S. Department of Justice, Bureau of Justice Statistics, 2004).

### Measures

#### Suicidal history

Two survey items which questioned prisoners on their lifetime history of suicidal thoughts (“Have you ever considered suicide?”) and attempted suicide (“Have you ever attempted suicide?”) were used to create three separate measures: (a) suicidal thoughts only; (b) attempted suicide; (c) suicidal thoughts/attempts. The first measure reflects prisoners who exclusively reported having ever considered suicide, excluding those who reported a previous suicide attempt (*n* = 1716). The second measure reflects prisoners who exclusively reported having previously made a suicide attempt (*n* = 2496). The third measure, a sum of the first two measures, reflects prisoners who reported having ever considered suicide and/or making a previous suicide attempt; thus, a global measure of ‘suicidal history’ (*n* = 4212). Each measure was binary coded (1 = yes, 0 = no).

#### Age

A quantitative measure of age in years (ranging from 16 to 84) was used, which was also squared (Age^2^) to examine the possibility of a curvilinear effect of age on the probability of reporting suicidal thoughts and behavior considering the nature and extent of these issues are expected to vary across the lifespan (Fiske & O’Riley, 2016). Following criterion for the classification of older prisoners (Grant, 1999; Morton, 1992), the continuous measure of age in years was also collapsed to reflect prisoners aged 49 years and younger (*n* = 16,278) and 50 years and older (*n* = 1907). The younger prisoner group was further collapsed into two strata: young-young (16 to 30 years of age; *n* = 6396) and old-young (31 to 49 years of age; *n* = 9882). On average, the sample was approximately 36 years of age (*SD* = 10.51) and younger prisoners (≤ 49 years of age) made up 89.5% of the total sample.

#### Sociodemographic characteristics

Female is coded as 1 (male = 0). Race/ethnicity was based on a series of dummy variables: Hispanic, White, Black, and ‘Other’ (i.e., Indigenous, Asian, Hawaiian/Pacific Islander, or other race/ethnicity). Education is a quantitative measure that captured the highest grade of school ever attended prior to the current incarceration, ranging from 0 to 18.

#### Psychological health and well-being

With respect to psychological disorders, prisoners were asked whether a mental health professional (e.g., a psychiatrist or psychologist) had ever told them they had: a depressive disorder; a bipolar or related disorder (i.e., manic-depression, bipolar disorder, or mania); schizophrenia or another psychotic disorder; posttraumatic stress disorder (PTSD); an anxiety disorder (e.g., panic disorder); a personality disorder (e.g., antisocial or borderline personality disorder). Each item was binary coded (1 = yes, 0 = no). A quantitative measure of psychological disorders was also created to reflect prisoners’ total number of reported diagnoses (ranging from 0 to 6). Measures of psychological symptoms reflect whether inmates had experienced symptoms consistent with hopelessness, sleep disturbance, schizophrenia/psychosis, and emotional dysregulation within the 12 months prior to data collection. Hopelessness is based on a single-item (yes/no) survey question which captured whether prisoners had given up hope for life or the future. Sleep disturbance is based on the aggregation of two survey items (yes/no) which captured whether prisoners had experienced a noticeable increase/decrease in the amount of time they slept, or if they had negative or frightening thoughts/dreams that made it difficult to sleep. Symptoms of schizophrenia/psychosis is based on the aggregation of four survey items (yes/no) which captured whether inmates had experienced auditory/visual hallucinations or bizarre delusions (e.g., believed people could read their mind or control their brain/thoughts). Emotional dysregulation is based on the aggregation of four survey items (yes/no) which captured whether inmates had lost their temper easily, been angry more often than usual, hurt or broken things in anger, or thought a lot about revenge on someone they were angry at. Each measure was binary coded (1 = yes, 0 = no). A quantitative measure of psychological symptoms was also created to reflect prisoners’ total number of reported symptoms, ranging from 0 to 11. Social disconnectedness was binary coded (1 = yes, 0 = no) and reflects whether prisoners had difficulty feeling close to friends/family.

#### Substance use

Bush, Shaw, Cleary, DelBanco, and Aronson’s (1987) 4-question screening test was used to identify prisoners with alcohol dependence (Cronbach’s *a* = .853; mean inter-item correlation = 0.591). Prisoners were asked whether, at any point in their life, they: felt they should cut down on drinking; had people annoy or criticize them about their drinking; felt bad or guilty about their drinking; had a drink first thing in the morning to steady nerves or get rid of a hangover. Two or more positive responses is indicative of alcohol dependence; thus, this measure was binary coded (1 = alcohol dependence, 0 otherwise). Prisoners were also asked about their lifetime history of illicit drug use for several different types of drugs (e.g., opiates, amphetamines, barbiturates, tranquilizers, crack/cocaine, phencyclidine, lysergic acid diethylamide or other hallucinogens, inhalants, etc.), which is a quantitative measure reflecting the number of different drugs used by prisoners, ranging from 0 to 15 listed categories of drugs.

#### Victimization

Sexual victimization is a categorical measure which reflects whether, before admission to prison, prisoners had ever been pressured or forced into any sexual contact against their will once, more than once, or not at all. Physical victimization reflects whether, before admission to prison, prisoners had ever been physically abused once, more than once, or not at all.

#### Physical illness and disability

Prisoners were asked about their lifetime history of physical health problems (e.g., cancer, paralysis, hypertension, stroke/brain injury, diabetes, heart problems, kidney problems, arthritis or rheumatism, asthma, cirrhosis of the liver, hepatitis, or a sexually transmitted disease other than AIDS), which was transformed into a binary variable to capture prisoners who reported at least one of the listed physical health problems (1 = yes, 0 = no). A quantitative measure was also created to reflect prisoners’ total number of reported physical health problems, ranging from 0 to 12. Similar to Stoliker and Galli (2019), disability in basic activities of daily living (hereafter, BADL disability) was coded as a binary variable (1 = yes, 0 = no) and reflects challenges in physical function, such as difficulty seeing (even when wearing glasses), difficulty hearing (even with a hearing aid), or requiring the use of aids to help with daily activities. A quantitative measure was also created to reflect prisoners’ total number of reported BADL disabilities, ranging from 0 to 3.

#### Interaction terms

To address the second hypothesis, six multiplicative terms were created to delineate the effect of age on suicidal thoughts and behavior when statistically adjusting for moderating effects of a third variable. These interaction effects are represented by formula (1).

1 $$ {\displaystyle \begin{array}{l}\ln \left[\frac{Pi}{1- Pi}\right]={\beta}_0+{\beta}_1\left(\mathrm{Female}=1\right)\dots +{\beta}_{27}\left(\mathrm{Age}\ast \mathrm{Hopelessness}\right)\\ {}+{\beta}_{28}\left({\mathrm{Age}}^2\ast \mathrm{Hopelessness}\right)+{\beta}_{29}\left(\mathrm{Age}\ast \mathrm{PhysicalIllness}=1\right)\\ {}+{\beta}_{30}\left({\mathrm{Age}}^2\ast \mathrm{PhysicalIllness}=1\right)+{\beta}_{31}\left(\mathrm{Age}\ast \mathrm{BADLdisability}=1\right)+{\beta}_{32}\left({\mathrm{Age}}^2\ast \mathrm{BADLdisability}=1\right)+e.\end{array}} $$

### Analytic procedure

**Fig. 1:**
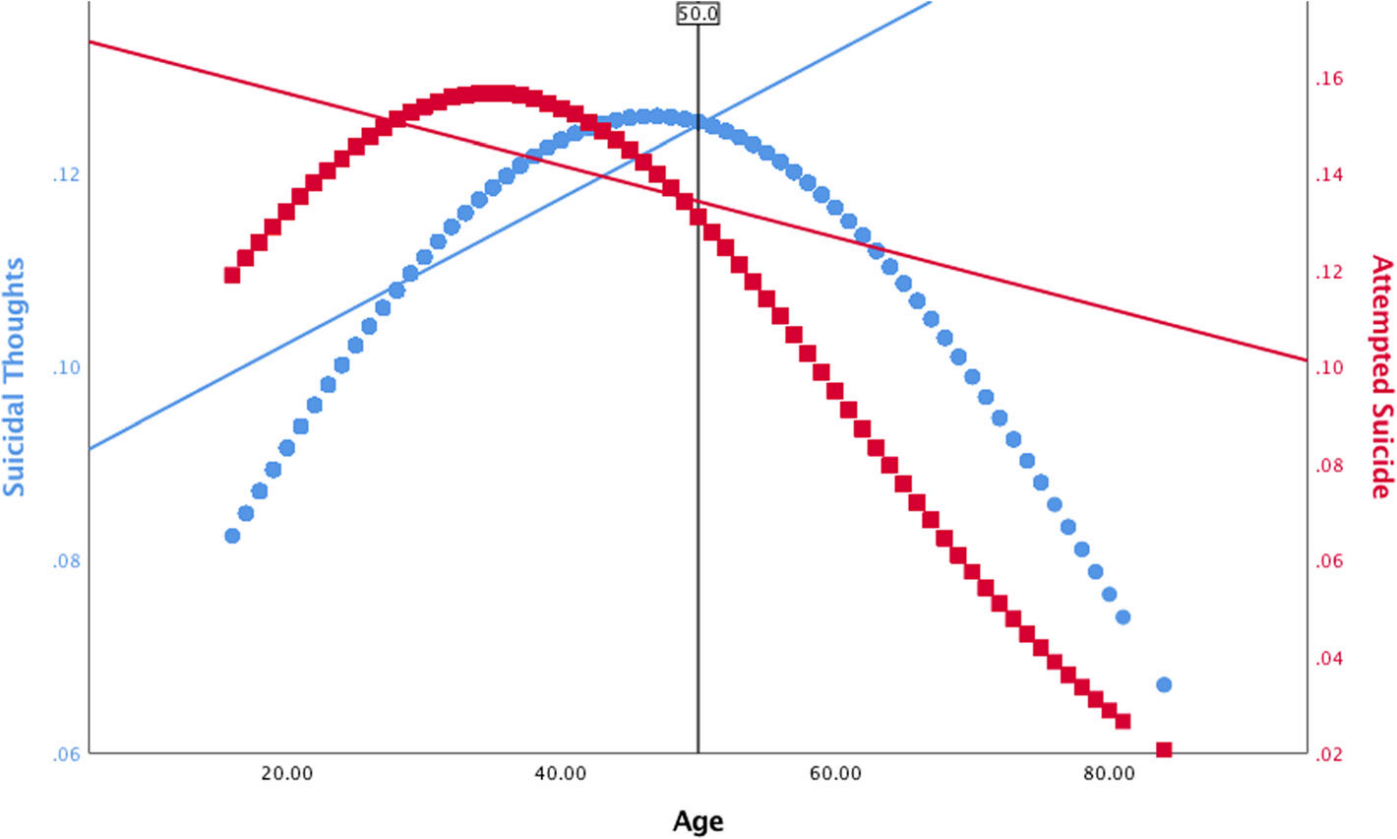
Predicted probability of reporting suicidal thoughts and attempted suicide across age, fitted with a linear regression line

**Fig. 2:**
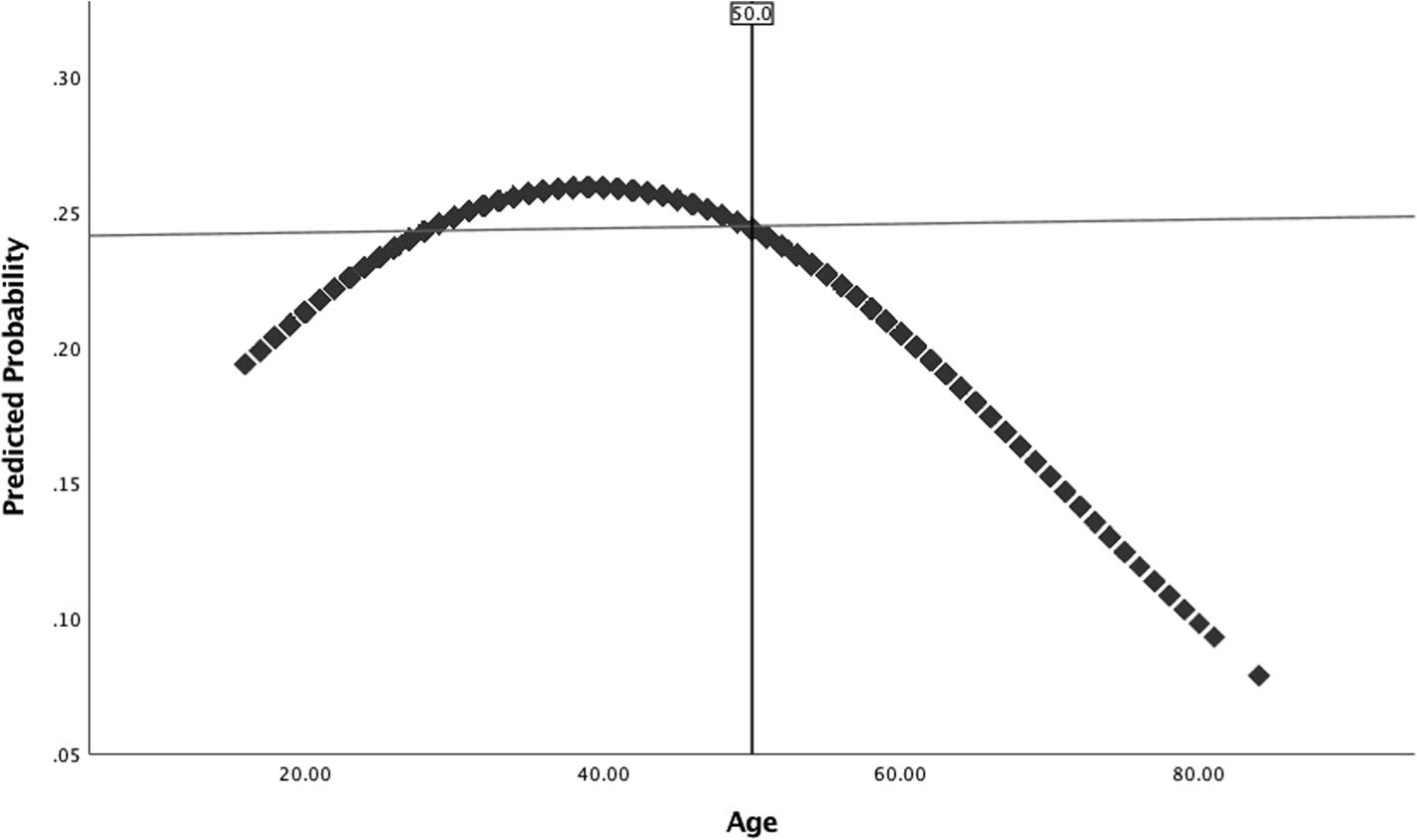
Predicted probability of reporting a suicidal history across age, fitted with a linear regression line

**Fig. 3:**
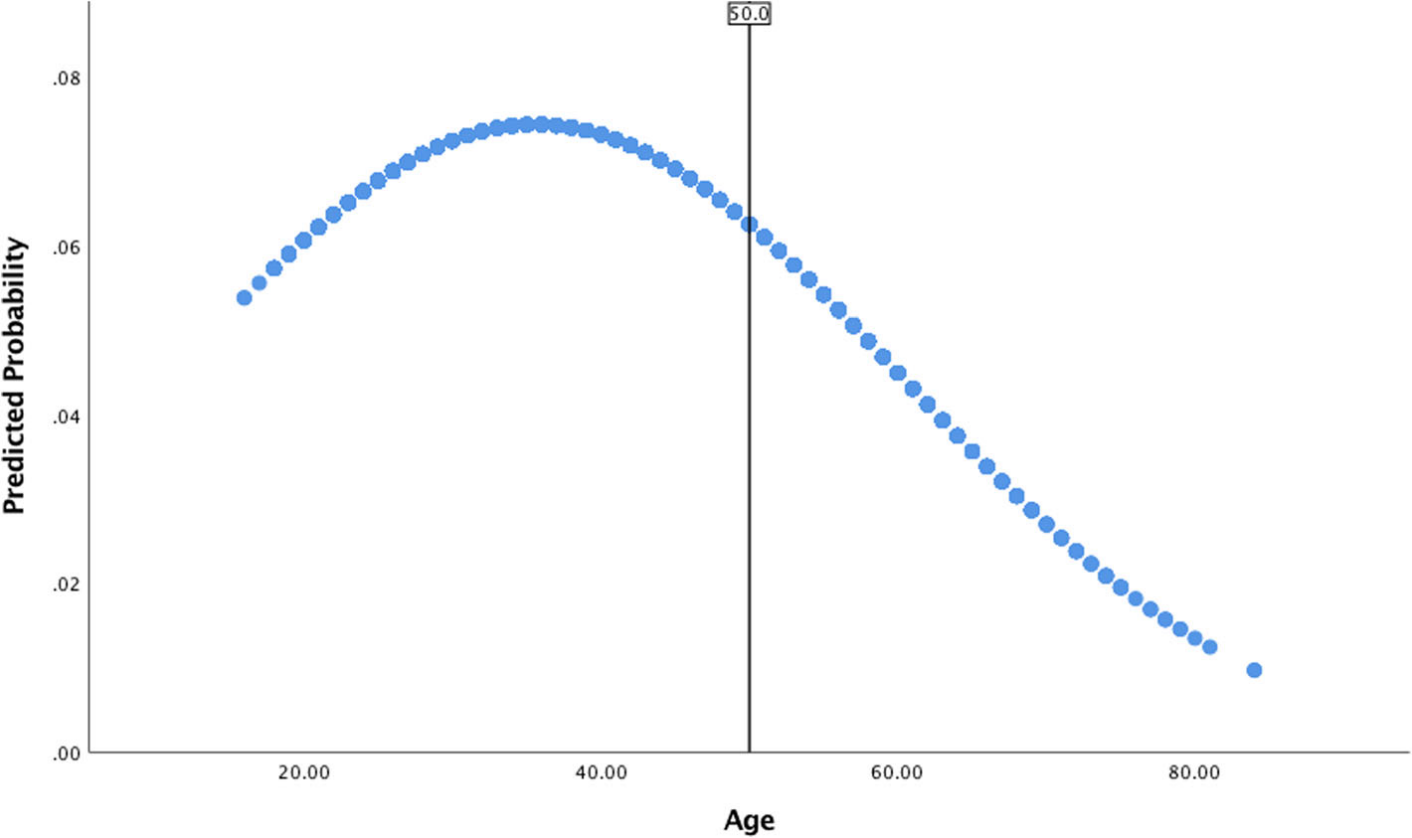
Predicted probability of reporting suicidal thoughts across age for prisoners with a reported physical illness, controlling for confounding effects

**Fig. 4:**
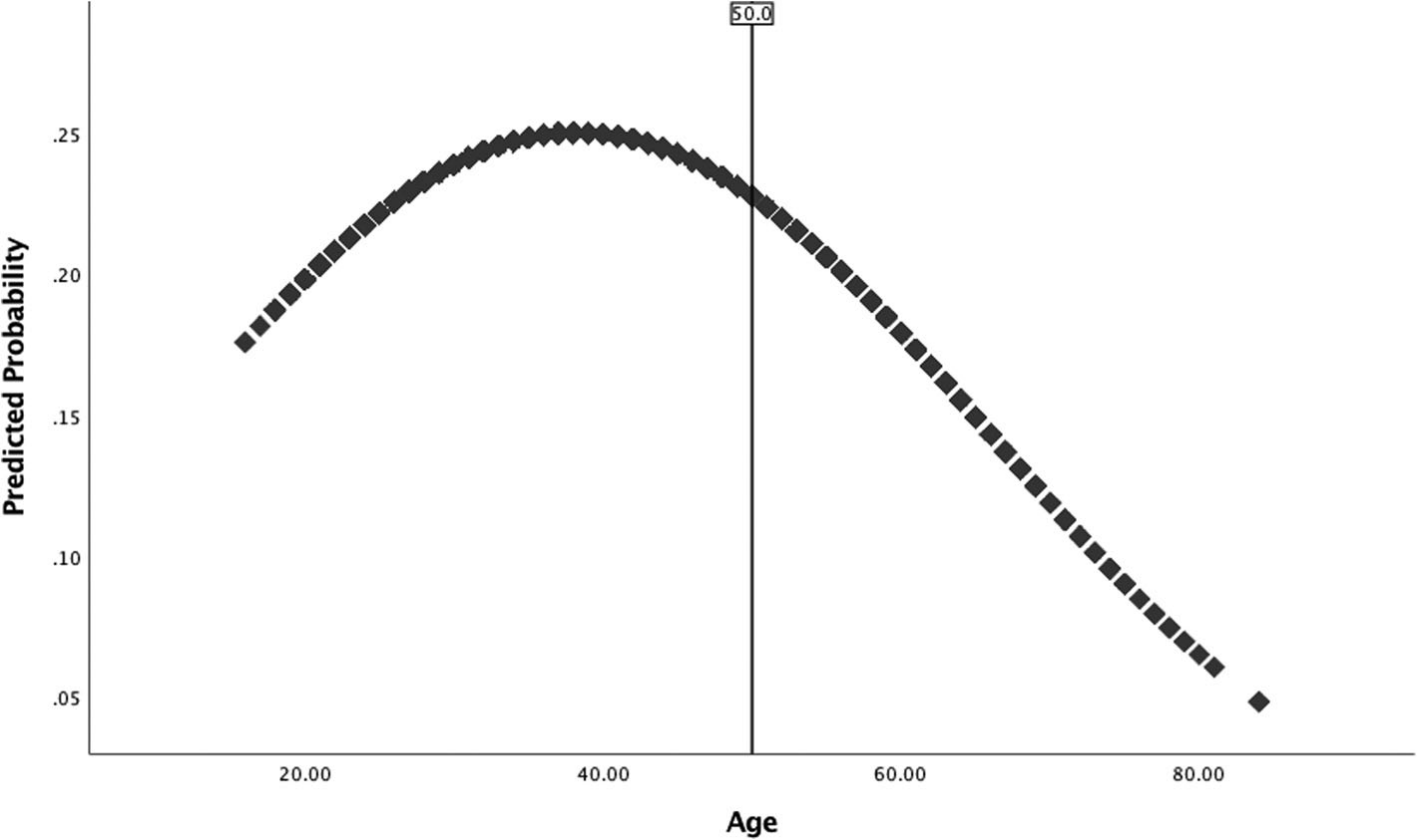
Predicted probability of reporting a suicidal history across age for prisoners with a reported physical illness, controlling for confounding effects

With the sample stratified according to age group (young-young, old-young, and older prisoners), analyses were run to estimate prevalence rates of suicidal thoughts and attempts (Table 1) as well as to provide summary statistics on the study measures according to those who reported a suicidal history (Table 2). Using the unstratified sample, bivariate binomial logistic regression analyses were conducted to estimate the unadjusted association between the quadratic effect of age (Age, Age^2^) and the suicidal outcome measures. Again, using the unstratified sample, multivariate binomial logistic regression models were created to estimate the main effects (Table 3) and interaction effects (Table 4) of predictors on each of the suicidal outcome measures. Models were also created to estimate the effects of predictors on the global measure of suicidal history separately for the older and younger prisoner sub-groups (Table 5). In this regard, a two-sample z-test (Paternoster, Brame, Mazerolle, & Piquero, 1998) was further applied to test whether log odds for pertinent predictor variables differ across younger and older prisoners. With respect to conducting multivariate analyses, consideration was given to the clustered nature of the data (i.e., inmates nested within 326 prisons) and Huber-White (robust) standard errors were used when estimating models to reduce bias in the standard errors of parameter estimates. Finally, several plots were created to highlight the predicted probability of reporting suicidal thoughts and/or behavior across age according to: (a) the bivariate relationship between the quadratic function of age (Age, Age^2^) and suicidal outcome measures (Figs. 1 and 2), and; (b) the relationship between the quadratic function of age (Age, Age^2^) and suicidal outcome measures, adjusting for moderating effects of factors expected to distinguish suicidal thoughts/behavior later as compared to earlier in life (Figs. 3 and 4). Overall, relatively few cases were missing from each predictor and criterion variable (between 0 and 2.1%) and listwise deletion was used when estimating multivariate models. All tests were two-tailed and probability values < 0.05 were considered statistically significant.

**Table 1:** Prevalence of suicidal thoughts and attempted suicide

	Total Sample (*N* = 18,185)	Younger Prisoners (*n* = 16,278)		Older Prisoners^a^ (*n* = 1907)
		Young-Young^b^ (*n* = 6396)	Old-Young^c^ (*n* = 9882)	
**In the Total Sample**				
Suicidal Thoughts Only	9.4%	8.6%	9.8%	10.4%
Adjusted Residual		−3.0	1.8	1.6
		$\mathcal{X}$^2^ (df = 2, *n* = 17,893) = 9.549, *p* = **.008**; Cramer’s *V* = 0.023		
Attempted Suicide	13.7%	13.9%	14.6%	8.7%
Adjusted Residual		.5	3.7	−6.8
		$\mathcal{X}$^2^ (df = 2, *n* = 17,899) = 47.455, *p* **< .001**; Cramer’s *V* = 0.051		
Suicidal Thoughts/Attempts	23.2%	22.5%	24.4%	19.1%
Adjusted Residual		−1.6	4.3	−4.4
		$\mathcal{X}$^2^ (df = 2, *n* = 17,891) = 27.401, *p* **< .001**; Cramer’s *V* = 0.039		
**Among Ideators/Attempters Only**^**d**^				
Suicidal Thoughts Only	40.7%	38.1%	40.2%	54.7%
Adjusted Residual		−2.5	−.8	5.7
Suicide Attempt	59.3%	61.9%	59.8%	45.3%
Adjusted Residual		2.5	.8	−5.7
Ratio^e^		1.62:1	1.48:1	0.83:1
		$\mathcal{X}$^2^ (df = 2, *n* = 4210) = 33.793, *p* **< .001**; Cramer’s *V* = 0.090		

^a^Aged 50 years and older

^b^16 to 30 years of age

^c^31 to 49 years of age

^d^*n* = 4212

^e^Ratio for number of attempts to number of suicidal thoughts only

**Table 2:** Sample characteristics according to total sample and suicidal history (stratified by age group)

	Total Sample (*N* = 18,185)	Suicidal History^a^		
		Young-Young^b^ (*n* = 1440)	Old-Young^c^ (*n* = 2408)	Older Prisoners^d^ (*n* = 364)
**Sociodemographic**				
Female = 1	21.4	35.1	33.1	29.1
Age (Quantitative)	35.83 (10.51)			
Dichotomous				
≤ 49	89.5			
≥ 50	10.5			
Multinomial				
16 to 30	35.2			
31 to 49	54.3			
50+	10.5			
Race/Ethnicity				
Hispanic	18.8	17.3	13.5	7.4
White	49.1	59.2	61.0	70.9
Black	42.5	32.8	33.5	25.3
Other	11.4	12.9	10.6	9.9
Education	10.95 (2.49)	10.58 (2.04)	11.02 (2.65)	11.82 (3.29)
**Psychological Disorder**				
Depressive	20.1	51.2	51.6	47.3
Bipolar/Related	10.5	30.8	31.6	20.1
Schizophrenia/Psychotic	4.3	10.1	14.1	10.2
PTSD	6.3	16.2	18.1	19.5
Anxiety	8.0	20.2	21.9	18.7
Personality	5.9	17.2	17.1	13.7
Mental Disorder (Quantitative)	0.56 (1.13)	1.45 (1.54)	1.54 (1.58)	1.30 (1.47)
**Psychological Symptoms**				
Hopelessness	6.7	17.8	16.9	22.8
Sleep Disturbance	45.6	75.5	65.6	60.2
Schizophrenia/Psychosis	11.4	28.8	25.7	21.2
Emotional Dysregulation	40.1	68.1	54.9	41.2
Total Symptoms (Quantitative)	1.71 (2.01)	3.52 (2.45)	2.78 (2.37)	2.22 (2.20)
**Social Disconnectedness**				
Not Feeling Close	29.3	57.4	45.6	35.4
**Substance Use**				
Alcohol Dependence	30.4	35.6	47.3	42.0
Number of Drugs Used	3.09 (3.25)	4.36 (3.65)	4.65 (3.80)	3.05 (3.61)
**Victimization**				
Sexual				
Never	86.0	69.7	68.3	73.1
Once	3.8	8.3	7.7	8.0
More than Once	8.5	21.5	22.9	17.6
Physical				
Never	79.0	55.8	57.6	66.8
Once	3.1	6.0	6.0	4.9
More than Once	16.4	37.9	35.8	27.2
**Physical Illness/Disability**				
Physical Illness = 1	55.9	61.3	76.5	88.2
Quantitative	1.12 (1.38)	1.13 (1.28)	1.79 (1.60)	2.82 (1.96)
BADL Disability = 1	15.9	16.4	24.9	45.1
Quantitative	0.19 (0.48)	0.19 (0.47)	0.32 (0.61)	0.65 (0.82)

*Note*. *BADL* Basic activities of daily living; data are presented as percentages (categorical data) or means with standard deviation in parentheses (continuous data)

^a^Inmates who reported suicidal thoughts/attempted suicide (*n* = 4212)

^b^16 to 30 years of age

^c^31 to 49 years of age

^d^Aged 50 years and older

**Table 3:** Multivariate binomial logistic regression predicting suicidal thoughts and behavior (main effects)

	Suicidal Thoughts Only^a^		Attempted Suicide		Suicidal Thoughts/Attempts	
	aOR (SE)	95% CI	aOR (SE)	95% CI	aOR (SE)	95% CI
Female = 1	.660 (0.086)	.557–.783***	1.32 (0.068)	1.15–1.51***	.960 (0.060)	.852–1.08
Age^b^	1.02 (0.016)	.987–1.05	1.01 (0.018)	.974–1.04	1.01 (0.013)	.986–1.04
Age^2b^	1.00 (0.0002)	.999–1.00	1.00 (0.0002)	.999–1.00	1.00 (0.0002)	.999–1.00
Race/Ethnicity						
Hispanic	.762 (0.089)	.639–.907**	.890 (0.078)	.763–1.04	.809 (0.064)	.712–.919**
White^c^	–	–	–	–	–	–
Black	.782 (0.068)	.684–.896***	.780 (0.063)	.688–.884***	.762 (0.052)	.689–.844***
Other	.787 (0.101)	.645–.960*	.977 (0.087)	.824–1.16	.866 (0.074)	.748–1.002
Education	1.06 (0.012)	1.03–1.08***	.960 (0.011)	.938–.982**	1.01 (0.009)	.991–1.03
Depressive Disorder	2.36 (0.082)	2.01–2.77***	2.79 (0.069)	2.43–3.20***	2.81 (0.060)	2.50–3.17***
Bipolar/Related Disorder	1.22 (0.105)	.995–1.50	2.03 (0.077)	1.75–2.37***	1.81 (0.075)	1.56–2.10***
Schizophrenia/Psychotic Disorder	1.04 (0.165)	.751–1.44	2.01 (0.112)	1.62–2.51***	1.69 (0.116)	1.34–2.12***
PTSD	1.10 (0.124)	.862–1.40	1.24 (0.095)	1.03–1.50*	1.29 (0.095)	1.07–1.55**
Anxiety Disorder	1.12 (0.109)	.906–1.39	1.07 (0.087)	.905–1.27	1.17 (0.084)	.996–1.39
Personality Disorder	1.20 (0.130)	.931–1.55	1.31 (0.096)	1.09–1.59**	1.37 (0.097)	1.13–1.66**
Hopelessness	2.46 (0.103)	2.01–3.02***	2.16 (0.089)	1.81–2.57***	2.77 (0.082)	2.36–3.26***
Sleep Disturbance	1.47 (0.065)	1.29–1.67***	1.28 (0.062)	1.13–1.45***	1.40 (0.049)	1.27–1.54***
Schizophrenic/Psychotic Symptoms	1.57 (0.089)	1.32–1.87***	1.54 (0.074)	1.33–1.78***	1.67 (0.068)	1.46–1.91***
Emotional Dysregulation	1.19 (0.065)	1.05–1.36**	1.09 (0.061)	.970–1.23	1.15 (0.049)	1.04–1.26**
Not Feeling Close	1.43 (0.065)	1.26–1.63***	1.16 (0.061)	1.03–1.31*	1.35 (0.050)	1.22–1.49***
Alcohol Dependence	1.19 (0.062)	1.06–1.35**	1.32 (0.057)	1.18–1.48***	1.28 (0.048)	1.16–1.40***
Number of Drugs Used	1.06 (0.008)	1.04–1.08***	1.03 (0.008)	1.01–1.04***	1.06 (0.007)	1.04–1.07***
Sexual Victimization						
Never^c^	–	–	–	–	–	–
Once	1.79 (0.137)	1.37–2.35***	1.78 (0.113)	1.43–2.23***	1.89 (0.104)	1.54–2.32***
More than Once	2.03 (0.109)	1.64–2.51***	2.09 (0.084)	1.77–2.46***	2.34 (0.080)	2.00–2.74***
Physical Victimization						
Never^c^	–	–	–	–	–	–
Once	1.57 (0.149)	1.17–2.10**	1.66 (0.131)	1.28–2.14***	1.74 (0.116)	1.39–2.19***
More than Once	1.77 (0.081)	1.51–2.08***	1.93 (0.068)	1.69–2.21***	2.00 (0.060)	1.78–2.25***
Physical Illness = 1	1.46 (0.064)	1.28–1.65***	1.34 (0.060)	1.19–1.51***	1.43 (0.048)	1.30–1.57***
BADL Disability = 1	1.05 (0.076)	.904–1.22	1.25 (0.069)	1.09–1.43**	1.16 (0.059)	1.03–1.30*
Constant	.013 (0.327)	.007–.025***	.048 (0.349)	.024–.094***	.047 (0.262)	.028–.079***
	*n* = 14,863		*n* = 17,230		*n* = 17,230	
Likelihood-ratio χ^2^_(26)_	1619.99***		3847.01***		5030.80***	
−2 Log Likelihood	8645.49		9923.17		13,589.88	
AIC	8699.49		9977.17		13,643.88	

*Note*. *BADL* Basic activities of daily living, *AIC* Akaike’s Information Criterion; models report adjusted odds ratios, Huber-White (robust) standard errors, and 95% confidence intervals

^a^Inmates who reported a suicide attempt are excluded from analysis

^b^Showing quadratic (curvilinear) effect of age

^c^Reference category

*p < .05, **p < .01, ***p < .001

**Table 4:** Multivariate binomial logistic regression predicting suicidal thoughts and behavior (interaction effects)

	Suicidal Thoughts Only^a^		Attempted Suicide		Suicidal Thoughts/Attempts	
	aOR (SE)	95% CI	aOR (SE)	95% CI	aOR (SE)	95% CI
**Hopelessness**						
Age x Hopelessness	0.942 (0.047)	0.858–1.03	0.944 (0.043)	0.866–1.03	0.948 (0.039)	0.878–1.02
Age^2^ x Hopelessness	1.001 (0.0006)	1.00–1.002	1.001 (0.0006)	1.00–1.002	1.001 (0.0005)	1.00–1.002
**Physical Illness**^**b**^						
Age x Physical Illness	1.06 (0.037)	0.990–1.15 ⊺	1.04 (0.043)	0.961–1.13	1.07 (0.032)	1.006–1.14*
Age^2^ x Physical Illness	0.999 (0.0005)	0.998–0.999*	1.00 (0.0006)	0.998–1.001	0.999 (0.0004)	0.998–0.999*
**BADL Disability**^**b**^						
Age x BADL Disability	1.02 (0.038)	0.945–1.10	0.959 (0.041)	0.884–1.04	0.995 (0.031)	0.935–1.06
Age^2^ x BADL Disability	1.00 (0.0005)	0.999–1.001	1.001 (0.0005)	1.00–1.002	1.00 (0.0004)	0.999–1.001
Constant	0.026 (0.545)	0.009–0.075***	0.052 (0.592)	0.016–0.165***	0.089 (0.442)	0.037–.212***
	*n* = 14,863		*n* = 17,230		*n* = 17,230	
Likelihood-ratio χ^2^_(32)_	1628.26***		3856.31***		5039.48***	
−2 Log Likelihood	8637.23		9913.87		13,581.19	
AIC	8703.23		9979.86		13,647.19	

*Note*. All main effect variables were entered into regression models—only interaction effects are presented as main effects are redundant; *AIC* Akaike’s Information Criterion; models report adjusted odds ratios, Huber-White (robust) standard errors, and 95% confidence intervals

^a^Inmates who reported a suicide attempt are excluded from analysis

^b^Binary-coded variable

⊺*p* < .10, *p < .05, **p < .01, ***p < .001

**Table 5:** Multivariate binomial logistic regression predicting suicidal history among older and younger prisoners separately

	Older Prisoners^a^		Younger Prisoners^b^	
	aOR (SE)	95% CI	aOR (SE)	95% CI
Female = 1	1.20 (0.206)	0.80–1.80	.951 (0.064)	0.84–1.08
Age	1.01 (0.014)	0.98–1.04	.998 (0.003)	0.99–1.00
Race/Ethnicity				
Hispanic	.407 (0.263)	0.24–0.68**	.856 (0.067)	0.75–0.98*
White^c^				
Black	.650 (0.180)	0.45–0.92*	.785 (0.055)	0.70–0.87***
Other	.735 (0.273)	0.43–1.25	.874 (0.078)	0.75–1.02
Education	1.01 (0.024)	0.96–1.05	1.01 (0.010)	0.99–1.03
Depressive Disorder	4.19 (0.205)	2.81–6.27***	2.72 (0.063)	2.40–3.08***
Bipolar/Related Disorder	.695 (0.303)	0.38–1.26	1.95 (0.078)	1.67–2.27***
Schizophrenia/Psychotic Disorder	1.70 (0.392)	0.79–3.67	1.72 (0.123)	1.35–2.18***
PTSD	2.32 (0.276)	1.35–3.98**	1.19 (0.101)	0.98–1.45
Anxiety Disorder	.987 (0.308)	0.54–1.80	1.20 (0.088)	1.01–1.42*
Personality Disorder	1.43 (0.362)	0.70–2.91	1.40 (0.101)	1.15–1.71**
Hopelessness	3.30 (0.236)	2.08–5.23***	2.72 (0.088)	2.29–3.23***
Sleep Disturbance	1.55 (0.166)	1.12–2.14**	1.38 (0.052)	1.24–1.53***
Schizophrenic/Psychotic Symptoms	2.06 (0.242)	1.29–3.32**	1.66 (0.071)	1.45–1.91***
Emotional Dysregulation	.962 (0.181)	0.67–1.37	1.16 (0.052)	1.05–1.29**
Not Feeling Close	1.33 (0.189)	0.92–1.93	1.35 (0.052)	1.22–1.50***
Alcohol Dependence	1.54 (0.160)	1.12–2.11**	1.26 (0.051)	1.14–1.39***
Number of Drugs Used	1.05 (0.022)	1.01–1.10*	1.06 (0.007)	1.05–1.08***
Sexual Victimization				
Never^c^				
Once	2.79 (0.365)	1.36–5.71**	1.81 (0.109)	1.47–2.25***
More than Once	3.62 (0.284)	2.07–6.32***	2.29 (0.083)	1.94–2.69***
Physical Victimization				
Never^c^				
Once	1.58 (0.396)	0.72–3.43	1.76 (0.122)	1.39–2.23***
More than Once	1.54 (0.212)	1.02–2.34*	2.05 (0.063)	1.81–2.32***
Physical Illness = 1	1.23 (0.217)	0.80–1.88	1.44 (0.050)	1.31–1.59***
BADL Disability = 1	1.27 (0.156)	0.93–1.73	1.13 (0.064)	1.00–1.29
Constant	.031 (0.895)	0.01–0.18***	.058 (0.159)	0.04–0.08***
	*n* = 1779		*n* = 15,451	
Likelihood-ratio χ^2^_(25)_	455.81***		4592.91***	

*Note*. *BADL* Basic activities of daily living; models report adjusted odds ratios, Huber-White (robust) standard errors, and 95% confidence intervals

^a^Aged 50 years and older

^b^Aged 49 years and younger

^c^Reference category

*p < .05, **p < .01, ***p < .001

## Results

### Descriptive and bivariate

As shown in Table 1, among the total sample, approximately 23% reported a history of suicidal thoughts and/or attempts, of which 9.4% accounts for a history of suicidal thoughts (only) and 13.7% accounts for a history of suicidal behavior. With respect to age strata, a suicidal history (i.e., suicidal thoughts/attempts) was slightly more prevalent among younger as compared to older prisoners; especially, the old-young stratum. Although a greater proportion of younger prisoners reported a previous suicide attempt, a slightly greater proportion of older prisoners reported a history of suicidal thoughts (only). These latter patterns are better reflected when assessing the subset of prisoners with a history of suicidal thoughts and/or behavior (i.e., among ideators/attempters only). Among the total subset, approximately 41% had reported suicidal thoughts (only) whereas roughly 59% had reported previously attempting suicide. With respect to age strata, for younger prisoners a history of suicidal behavior was more commonly reported than a history of suicidal thoughts (only), whereas for older prisoners a history of suicidal thoughts (only) was more commonly reported than a history of suicidal behavior (see Table 1).

As shown in Table 2, across all age groups, a psychological disorder diagnosis and symptoms of poor psychological health were more common among those with a suicidal history as compared to the sample as a whole. Findings suggest that hopelessness is more prevalent among older prisoners with a suicidal history as compared to their younger counterparts [$\mathcal{X}$^2^ (df = 2, *n* = 4179) = 7.80, *p* = .020; Cramer’s *V* = 0.043]. Prevalence rates for depressive disorder did not significantly differ across younger and older prisoners with a suicidal history [$\mathcal{X}$^2^ (df = 2, *n* = 4199) = 1.97, *p* = .372; Cramer’s *V* = 0.022]. Table 2 also shows that social disconnectedness, substance abuse, sexual and physical victimization, poor physical health, and BADL disability were more common among those with a suicidal history as compared to the sample as a whole. Results further suggest that, as compared to their younger counterparts, older prisoners with a suicidal history showed a higher prevalence of physical illness [$\mathcal{X}$^2^ (df = 2, *n* = 4195) = 156.27, *p* < .001; Cramer’s *V* = 0.193], as well as a higher average number of physical illnesses [Welch-Statistic (df = 2, *n* = 4211) = 177.94, *p* < .001; Eta squared = 0.093]. Similarly, as compared to their younger counterparts, older prisoners with a suicidal history showed a much higher prevalence of BADL disability [$\mathcal{X}$^2^ (df = 2, *n* = 4203) = 137.81, *p* < .001; Cramer’s *V* = 0.181], as well as a higher average number of BADL disabilities [Welch-Statistic (df = 2, *n* = 4212) = 64.69, *p* < .001; Eta squared = 0.041]. In general, effect size statistics for these bivariate associations suggest relatively small to medium effects.

Bivariate logistic regression analyses revealed a statistically significant association between the quadratic effect of age and history of suicidal thoughts (Age: OR = 1.05 [95% CI: 1.02, 1.08], *SE* = 0.014, *p* = 0.001; Age^2^: OR = 0.999 [95% CI: 0.9991, 0.9998], *SE* = 0.0002, *p* = 0.004), attempted suicide (Age: OR = 1.06 [95% CI: 1.04, 1.09], *SE* = 0.014, *p* < 0.001; Age^2^: OR = 0.999 [95% CI: 0.9987, 0.9994], *SE* = 0.0002, *p* < 0.001), and suicidal history (Age: OR = 1.05 [95% CI: 1.03, 1.08], *SE* = 0.010, *p* < 0.001; Age^2^: OR = 0.999 [95% CI: 0.9990, 0.9995], *SE* = 0.0001, *p* < 0.001). This suggests a curvilinear relationship between age and the probability of reporting suicidal thoughts and behavior. Figures 1 and 2 further illustrate these patterns. Figure 1 highlights that examining the relationship between age and suicidal thoughts or behavior as a linear function would have led to some misleading conclusions, as the linear regression line oversimplifies the associations to suggest that an increase in age corresponds to an increase in the probability of reporting suicidal thoughts and a decrease in the probability of reporting a previous suicide attempt. Instead, Fig. 1 shows that an increase in age corresponds to a higher probability of reporting suicidal thoughts, peaking around late-40s with a decline in the probability of reporting suicidal thoughts with an increase in age thereafter. Figure 1 also shows that an increase in age corresponds to a higher probability of reporting a previous suicide attempt, peaking around the mid-30s with a decline in the probability of reporting a previous suicide attempt with an increase in age thereafter. Figure 2 shows the association between age and the global measure of suicidal history, indicating that the probability of reporting suicidal thoughts and/or attempts peaks around 40 years of age and declines with an increase in age thereafter.

### Multivariate

Results from multivariate main-effects models (Table 3) highlight several factors that are significantly associated with odds of reporting suicidal thoughts (only) and a previous suicide attempt. Prisoners at increased odds of reporting suicidal thoughts also reported a depressive-disorder diagnosis, feelings of hopelessness, sleep disturbance, schizophrenic/psychotic symptoms, emotional dysregulation, social disconnectedness, alcohol dependence, a greater number of different drugs used, sexual and physical victimization (once or more than once, compared to never), as well as at least one of the listed physical health problems. Similar patterns were found when estimating odds of reporting a previous suicide attempt, with exception to the fact that prisoners at increased odds of reporting a suicide attempt also reported a diagnosis for a bipolar or related disorder, schizophrenia or another psychotic disorder, PTSD, a personality disorder, as well as a BADL disability; emotional dysregulation was not significantly associated with a previous suicide attempt. Importantly, results from Table 3 show that associations between Age, Age^2^, and the suicidal outcome measures are no longer statistically significant when adjusting for known correlates of suicidal thoughts and behavior. This might suggest that age-based variation in suicidal thoughts and/or behavior is a function of other relevant explanatory factors.

The use of multiplicative terms aids in elucidating whether the relationship between age and suicidal thoughts and/or attempts is moderated by other explanatory factors, such as those expected to distinguish suicidal thoughts and behavior later as compared to earlier in life. Results from multivariate models estimating interaction effects (Table 4) suggest that the effect of poor physical health on the probability of reporting a suicidal history, and suicidal thoughts more specifically, is a function of age. Figures 3 and 4 further illustrate these patterns. Figure 3 shows that the probability of reporting suicidal thoughts for prisoners with a physical health problem increases with age, peaking around mid- to late-30s with a decline in the probability of reporting suicidal thoughts with an increase in age thereafter. It should be noted, however, that the moderating effect of physical illness on Age, Age^2^ and suicidal thoughts is only nearing significance. Figure 4 shows that the probability of reporting a history of suicidal thoughts and/or attempts for prisoners with a physical health problem increases with age, peaking around 40 years of age with a decline in the probability of reporting a suicidal history with an increase in age thereafter. Moderating effects of hopelessness and BADL disability on the relationship between Age, Age^2^ and suicidal outcome measures were not statistically significant (Table 4).

Results from the multivariate model(s) estimating the effects of predictors on the global measure of suicidal history, separately for older and younger prisoners (Table 5), revealed that older prisoners at increased odds of reporting a suicidal history also reported a depressive disorder diagnosis, PTSD, feelings of hopelessness, sleep disturbance, schizophrenic/psychotic symptoms, alcohol dependence, a greater number of different drugs used, sexual victimization once or more than once (compared to never), and physical victimization more than once (compared to never). Notably, a depressive disorder diagnosis and feelings of hopelessness, as well as sexual victimization, were relatively strong predictors of a suicidal history among older prisoners. Poor physical health and BADL disability were not statistically significant predictors of a suicidal history among older prisoners.

To test the possibility that, among older prisoners, the relationship between poor physical health and the suicidal outcome measures is mediated by depression and/or hopelessness, Baron and Kenny’s (1986) method was used. It was found that poor physical health (binary-coded) was significantly and positively associated with depressive disorder (OR = 2.04 [95% CI: 1.40, 2.98], *SE* = .192; *p* < .001), attempted suicide (OR = 3.46 [95% CI: 1.86, 6.47], *SE* = .318; *p* < .001), and the global measure of suicidal history (OR = 2.03 [95% CI: 1.44, 2.88], *SE* = .178; *p* < .001) but not suicidal thoughts (OR = 1.45 [95% CI: 0.97, 2.19], *SE* = .208; *p* = .072). When attempted suicide was regressed on both poor physical health and depressive disorder, depressive disorder maintained a strong effect (OR = 7.78 [95% CI: 5.53, 10.95], *SE* = .174; *p* < .001) while the effect of poor physical health was statistically weakened (OR = 2.82 [95% CI: 1.49, 5.35], *SE* = .326; *p* = .001). The same effect is found when the global measure of suicidal history was regressed on depressive disorder (OR = 9.04 [95% CI: 6.87, 11.89], *SE* = .140; *p* < .001) and poor physical health (OR = 1.68 [95% CI: 1.16, 2.44], *SE* = .190; *p* = .006). This suggests that, among older prisoners, the association between physical health problems and attempted suicide, as well as a suicidal history in general, is partially mediated by depression. No statistically significant results were found when assessing the mediating effects of hopelessness on the relationship between poor physical health and the suicidal outcome measures.

We further tested the possibility that, among older prisoners, the relationship between BADL disability and the suicidal outcome measures is mediated by depression and/or hopelessness. It was found that BADL disability (binary-coded) was significantly and positively associated with depressive disorder (OR = 2.07 [95% CI: 1.61, 2.66], *SE* = .127; *p* < .001), suicidal thoughts (OR = 1.64 [95% CI: 1.21, 2.22], *SE* = .155; *p* = .001), attempted suicide (OR = 2.33 [95% CI: 1.68, 3.22], *SE* = .165; *p* < .001), and the global measure of suicidal history (OR = 1.97 [95% CI: 1.56, 2.50], *SE* = .120; *p* < .001). When suicidal thoughts was regressed on both BADL disability and depressive disorder, depressive disorder maintained a strong effect (OR = 7.11 [95% CI: 5.08, 9.96], *SE* = .172; *p* < .001) while the effect of BADL disability lost significance completely (OR = 1.35 [95% CI: 0.98, 1.88], *SE* = .166; *p* = .067). This suggests that, among older prisoners, the association between BADL disability and suicidal thoughts is fully mediated by depression. There were no statistically significant mediating effects of depression on the associations between BADL disability and attempted suicide and the global measure of suicidal history. In addition, no statistically significant results were found when assessing the mediating effects of hopelessness on the relationship between BADL disability and the suicidal outcome measures.

Furthermore, results from the multivariate model(s) estimating the effects of predictors on the global measure of suicidal history, separately for older and younger prisoners, revealed that younger prisoners showed patterns somewhat similar to older prisoners; however, there are also some differences between these groups (Table 5). Aside from those associations similar to older prisoners, younger prisoners at increased odds of reporting a suicidal history also reported a bipolar or related disorder, schizophrenia or another psychotic disorder, an anxiety disorder, a personality disorder, emotional dysregulation, and social disconnectedness. Notably, poor physical health was significantly and positively associated with reporting a suicidal history among younger prisoners. Conversely, PTSD was significantly (and positively) associated with reporting a suicidal history for older, but not younger, prisoners. For younger prisoners, a depressive disorder diagnosis and feelings of hopelessness were relatively strong predictors of a suicidal history (much like older prisoners). Based on models from Table 5, we further tested whether logistic regression coefficients for pertinent explanatory variables significantly differed for younger and older prisoners (using a two-sample z-test). Analyses suggest that depressive disorder is a significantly stronger predictor for older as compared to younger prisoners (z = − 2.02, *p* = 0.043); however, no statistically significant differences were found when assessing regression coefficients for hopelessness (z = − 0.75, *p* = 0.448), poor physical health (z = 0.72, *p* = 0.470), and BADL disability (z = − 0.67, *p* = 0.501).

## Discussion

There has been a marked lack of research which adopts an age-focused analysis of suicidality among prisoners, despite a growing body of literature suggesting (1) there are important differences in suicidal ideation and behavior later as compared to earlier in the lifespan; (2) that rates of suicide-related deaths are highest among older adults (including prisoners), and; (3) the trend towards aging prison populations in high-income countries (Barry et al., 2017; Fiske & O’Riley, 2016; Lutz et al., 2016; O’Riley et al., 2014; Stanley et al., 2016). The purpose of the current study was to investigate the relationship between age and suicidal thoughts and attempts among a sample of prisoners, with particular focus on factors that may explain any age-based variability in these suicidal outcome measures. Two hypotheses were proposed: (a) incidence of suicidal thoughts and attempts will be greater among older as compared to younger prisoners; (b) age-based variability in suicidality is a function of factors that are expected to distinguish suicidal thoughts/behavior later as compared to earlier in life. In light of these aims, several research and practical implications can be extracted from this study.

Although prevalence rates showed that, among the total sample (*N* = 18,185), a history of suicidal thoughts and attempted suicide was not the norm, younger prisoners more commonly reported a suicidal history as compared to older prisoners (particularly, the ‘old-young’ stratum). Interestingly, however, analyses of the subset of prisoners with a suicidal history (*n* = 4212) highlighted that younger prisoners were more likely to have engaged in suicidal behavior (particularly, the ‘young-young’ stratum), whereas older prisoners were more likely to have experienced suicidal thoughts only. This pattern for older prisoners is interesting given that community-based research suggests suicidal ideation is *less* commonly reported among older adults (Lutz et al., 2016) and that older adults are *less* likely to express/report suicidal ideation prior to death (Fiske & O’Riley, 2016). As such, it is plausible that prevalence rates for suicidal ideation identified among older prisoners in the current study are still conservative.

Analyses further showed a curvilinear relationship between age and the probability of reporting suicidal thoughts and/or behavior. In this case, the probability of reporting suicidal thoughts peaked around late-40s, whereas the probability of reporting attempted suicide peaked around mid-30s—both following a downward trend thereafter (Fig. 1). There are two plausible explanations for these curvilinear patterns. The first possibility is that suicidal thoughts and attempted suicide genuinely constitute a more prominent issue among prisoners who are around the middle of their lifespan. In line with this notion, there is a body of evidence that suggests older, and aging, adults generally have better psychological health as compared to those earlier in the lifespan (Fiske & O’Riley, 2016; Van Orden & Conwell, 2016). Future research should examine this among prison populations. Secondly, there is the possibility that data reflect a ‘selection’ effect. In this case, downward trends in the predicted probability of reporting suicidal thoughts and/or behavior among older prisoners could be explained by the lethality of suicidal thoughts/behaviors in later life. Older adults are more likely than younger individuals to complete suicide on the first attempt, owing to the use of more violent methods, having a greater determination to die from an attempt, and presenting fewer opportunities to be rescued as a result of physical frailty (Conwell, 2013; Conwell et al., 1998; Conwell et al., 2002; Institute of Medicine, 2002; Stanley et al., 2016). Therefore, curvilinear patterns observed in the present sample may reflect a selection bias, whereby individuals who make a fatal first attempt at suicide later in life are effectively ‘selected’ out of the sample. It is also conceivable that, if the propensity for suicidal thoughts and actions is in fact greatest among prisoners around the middle of their lifespan, then there is also the potential that some who possess risk for suicide will not ‘enter’ into older age (again, suggesting a selection bias).

Drawing from the above findings, prison administrators should consider allocating resources to the identification, assessment, and treatment of prisoners at risk of suicidal ideation and behavior across the lifespan, but especially around young adulthood and mid-life. For younger prisoners, it would be appropriate to target behavioral risk and the associated precipitating factors; in this effort, prison administrators and mental health professionals should acknowledge both individual- and prison- level contributory factors (see Stoliker, 2018; Stoliker et al., 2020). Furthermore, given the high lethality of attempts among older individuals, along with the challenges imposed by older individuals’ reluctance to express suicidal thoughts, it is especially important to implement routine screening of older prisoners to identify the nature and extent of suicidal risk. This should be done at reception and throughout the individual’s sentence (Gooding et al., 2017) using validated instruments (e.g., the Geriatric Suicide Ideation Scale [GSIS], Heisel & Flett, 2016; the Scale for Suicidal Ideation, Beck, Kovacs, & Weissman, 1979). The identification of suicidal risk among older individuals becomes increasingly difficult as it may be somewhat common for thoughts of death to surface as a result of the aging process; however, wishing for death in later life is not an adaptive response to aging and age-related stressors (Van Orden & Conwell, 2016). Researchers and clinicians should therefore aim to differentiate between those who think about death as a ‘normal’ part of the aging process and those who have a desire for death. At any rate, targeting suicidal ideation among older prisoners may be crucial for reducing the likelihood of a suicidal event.

From a lifespan developmental perspective (Fiske & O’Riley, 2016), it is suggested that individuals who are unable to cope with, and adapt to, age-related adversities and limitations (e.g., physical and cognitive limitations, interpersonal loss, etc.) will be at increased risk of suicidal ideation. Those with a desire to die owing to their inability to cope with, and adapt to, life’s changes, combined with the capability for suicide (i.e., a lowered fear of death and higher pain tolerance), may also be at risk for engaging in suicidal behavior (Fiske & O’Riley, 2016; Joiner, 2005; Klonsky & May, 2015; Van Orden et al., 2010). Accordingly, it was expected that several empirically established age-associated factors may distinguish older and younger prisoners who experience suicidal thoughts and/or have attempted suicide, such as depression/hopelessness (Conwell et al., 1991; Conwell et al., 2002), physical health problems (Duberstein et al., 2004, b; Erlangsen et al., 2005; Lutz et al., 2016), and BADL disability (Barry et al., 2017; Conwell et al., 2010; Dennis et al., 2009).

Bivariate analyses highlighted that hopelessness, physical health problems, and BADL disability were more common among older prisoners with a suicidal history as compared to their younger counterparts. Investigation into the moderating effects of these factors provides further insight into age-related patterns (Table 4). Though there were not many statistically significant effects, it was found that the probability of reporting a suicidal history among those with a physical health problem was greater for younger as compared to older prisoners (see Fig. 4). This is consistent with prior evidence suggesting the association between illnesses and suicidal ideation is stronger for younger compared to older adults (Scott et al., 2010). Therefore, researchers and mental health professionals must be cognizant of the fact that just because aging is associated with the experience of poorer physical and functional health does not necessarily mean health conditions differentiate risk of suicide later as compared to earlier in the lifespan. This also lends credibility to the hypothesis that the inability to cope with, and adapt to, age-related adversities and limitations may be more pronounced when encountered earlier as opposed to later in the lifespan. As previously mentioned, prisoners often display an ‘accelerated’ physical age and experience physiological diseases/illnesses much earlier in the lifespan as compared to their non-incarcerated counterparts (Barry et al., 2017; Grant, 1999; Kratcoski & Pownall, 1989; Maschi et al., 2013; Reviere & Young, 2004; Williams et al., 2006). Consequently, these individuals might be faced with the challenge of coping with, and adapting to, these adversities earlier in the lifespan than might otherwise be expected. Taken together, coping and adaptation may be the key predictive construct(s) (Fiske & O’Riley, 2016; see also Conwell et al., 2002; Duberstein et al., 1994). Future research should therefore investigate whether suicidal risk among prisoners is linked to their ability to cope with, and adapt to, significant life changes (especially those associated with aging). Considering older offenders may be sentenced to imprisonment for the first time in older age (see a Canadian study by Uzoaba, 1998), and that this type of offender is likely to experience challenges adjusting to prison life (Morton, 1992), researchers should also focus on older prisoners’ ability to cope with, and adapt to, prison life as an explanation for suicidal thoughts and behavior.

While results from the current study suggest that physical illness was a unique and positive predictor of suicidal history for younger prisoners, ad hoc tests revealed that the effect of physical illness and disability may be slightly more complex for older prisoners. In particular, the effect of physical illness/disability on suicidal history among older prisoners is, in part, explained through its influence on depression (consistent with Lutz et al., 2016). In line with this finding, using the same data as the current study Stoliker and Galli (2019) present evidence to suggest that in a multivariate context BADL disability, as well as certain physical health issues, uniquely predict depressive disorder/symptoms among older prisoners. Multivariate analyses from the present study showed that depression/hopelessness were relatively strong predictors of a suicidal history among older prisoners and, further, the effect of depressive disorder was significantly stronger for older as compared to younger prisoners—supporting evidence that suggests depressive symptomatology is more common among older suicide victims (Conwell et al., 2002). Considering these findings, and that depression is common among older prisoners in general (see Stoliker & Galli, 2019), prison administrators should aim to implement effective strategies for identifying, assessing, and treating older prisoners experiencing depressive symptomatology to reduce the potential precipitating effects of this negative affective state on suicidal ideation and behavior. Researchers should further explore the underlying mechanisms of psychiatric illness among older prisoners, especially precipitators of depression/hopelessness, which may elucidate pathways to suicidal thoughts and actions for this demographic. In this effort, it would be important to assess how psychiatric illness may be linked to stresses and adversities of prison life (see Barry et al., 2017). In any case, prisoners of all ages should be receiving adequate physical health services, and mental health treatment should target any stresses related to physical health problems (Stoliker & Galli, 2019; Stoliker & Varanese, 2017).

### Limitations

Findings should be interpreted in light of several limitations. First, it is important to acknowledge the sampling bias induced by suicide fatalities. Considering that suicidal behaviors are more lethal with increasing age (Stanley et al., 2016), findings from the current study may be biased as data only captured non-fatal attempters. Second, the use of lifetime historical accounts of suicidal thoughts and attempted suicide presents a major limitation, as there is no clear indication as to the exact point in the lifespan that suicidal thoughts and/or behavior took place (or if it occurred multiple times over the life course) only the age at which it was reported. Ideally, future research should capture the specific age at which suicidal thoughts/attempts occurred to better test the hypotheses in this study. Relatedly, because outcome measures were based on lifetime history we were unable to parse-out suicidal thoughts and attempts that occurred prior to, or during, incarceration. As such, we excluded empirically relevant criminological and institutional factors from our analyses. Research that captures incidence of suicidal thoughts and behavior in the prison setting would be in better position to estimate the effects of criminological and institutional factors. In this case, the nature of an offender’s legal situation and sentence, as well as experiences of prison life (Stoliker, 2018; Stoliker et al., 2020), may be important predictors of suicidal ideation and behavior. Third, the self-report nature of the survey also presents some limitation as prisoners may be unwilling to provide accurate responses on sensitive items (Favril et al., 2020), despite being informed that responses would be kept confidential.

Fourth, the survey used single-item measures of suicidal thoughts and attempts. These measures lack important detail on suicidal ideation and behavior given that individuals may experience thoughts about ending their life which vary in degree of severity (Klonsky & May, 2015) as well as vary in the lethality of, and intent to die from, an attempt (Magaletta et al., 2008). Future research should therefore include measures which capture the severity of suicidal ideation (e.g., the Scale for Suicidal Ideation: Beck et al., 1979) and the lethality of, and intent to die from, an attempt (see Magaletta et al., 2008). Fifth, the survey data were collected in 2004 and, therefore, it is likely that findings do not necessarily reflect current trends among U.S. prisoners—especially considering the number of older prisoners has substantially grown since the data collection period (see Stoliker & Galli, 2019). In addition, the cross-sectional nature of the data limits any assumption of causal order between explanatory and outcome variables. Finally, while we found that several explanatory variables were statistically significant predictors of suicidal thoughts/behavior some of the effect sizes were small (OR closer to 1.0) and, therefore, these may be of little practical significance.

## Conclusions

This was one of very few studies to investigate suicidal thoughts and behavior among prisoners from a perspective intended to increase insight into age-related patterns. Considering that late-life suicidal ideation and behavior is expected to differ from suicidal ideation and behavior earlier in the lifespan, along with findings from the present study which provide preliminary evidence to suggest that younger and older prisoners may exhibit differing patterns of suicidal risk, prison administrators should consider incorporating a lifespan developmental approach to suicide prevention. That is, a prevention strategy which acknowledges differences in the nature of suicidal thoughts and behavior across the lifespan. In any case, the successful implementation of suicide prevention strategies in prison is predicated on a senior-management style that prioritizes suicide prevention and offers emotional and practical support to staff in suicide prevention efforts (Slade & Forrester, 2015). This will assuredly require a shift in the occupational roles of all correctional staff to include initiatives for managing and reducing inmate suicidality (Forrester & Slade, 2014; Marzano et al., 2016; Slade & Forrester, 2015; World Health Organization (WHO), 2007).

Reducing suicide among older adults may come with additional challenges, requiring upstream prevention strategies such as targeting ageism and promoting positive perceptions and attitudes about older adults and aging (Van Orden & Deming, 2018). Prison administrators should therefore aim to identify and reduce ageism in the prison setting as this may be an important first step in the process toward connecting older prisoners at risk of suicide to appropriate resources, such as geriatric medicine. Van Orden and Deming (2018) contend that high-quality geriatric medicine likely functions as suicide prevention for older and aging individuals, as geriatricians are trained to promote physical and cognitive functioning, as well as focus on better overall well-being as a goal. A major challenge, however, is the low number of geriatricians available to the general public and the difficulty of attracting gerontology specialists into prison medical systems. Health-care personnel working in the prison setting (e.g., physicians and mental health professionals) will therefore need to target a constellation of risk factors for suicidal thoughts/behavior among older prisoners, including psychiatric illness (especially depressive symptomatology), substance use, victimization, in addition to physical well-being. This will likely necessitate specialized training in geriatric care, which is certainly an enterprise worth pursuing given the aging prison population in the U.S. (Barry et al., 2017; Blowers & Blevins, 2015; Stoliker & Galli, 2019).

All things considered, there are still many questions that remain. While community-based research on late-life suicidal thoughts and behavior is progressing, little progress has been made in this area of research with respect to the prison context. More research is needed on age-based variability in suicidal thoughts and attempted suicide among prisoners, as well as the factors that might explain this variability. In these efforts, researchers should utilize Fiske and O’Riley’s (2016) lifespan developmental theory as a framework for investigating how suicidal thoughts and behavior vary across age and whether age-based variability is associated with an inability to cope with, or adapt to, life’s challenges (especially those associated with aging). It is imperative to address these gaps in order to distinguish the nature and risk of suicidal thoughts and behavior later as compared to earlier in the lifespan and, ultimately, develop effective suicide prevention strategies to manage suicidal thoughts and risk of attempted suicide among prisoners.

## Data Availability

Data analyzed for the current study are available in the Inter-university Consortium for Political and Social Research (ICPSR) repository, retrieved from: 10.3886/ICPSR04572.v6
